# Microfracture of Acetabular Rim After Segmental Labral Resection to Restore the Morphology and Function of Labrum: A Retrospective Study of More than 2 Years Follow‐up

**DOI:** 10.1111/os.13131

**Published:** 2021-10-18

**Authors:** Tiao Su, Jing Li, Liu Yang, Guang‐xing Chen

**Affiliations:** ^1^ Center for Joint Surgery Army Medical University Chongqing China; ^2^ Radiology Department, Southwest Hospital Army Medical University Chongqing China

**Keywords:** 3D DESS, Hip arthroscopy, Irreparable labral lesion, Marrow stimulation, Microfracture

## Abstract

**Objective:**

To report on the clinical outcome of patients undergoing combined arthroscopic treatment of labral resection and microfracture at the rim of acetabulum at a minimum 2‐year follow‐up.

**Methods:**

The retrospective study included 38 patients undergoing hip arthroscopy for irreparable labral injury from 24 February 2014 to 26 February 2018. Thirteen patients were excluded owing to patient refusal of participation and concomitant diseases like synovial chondromatosis and dysplasia hip. The study group consisted of patients undergoing combined arthroscopic labral resection and microfracture at the rim of acetabulum (MICRO Group: 20 patients), arthroscopic labral resection alone (RESEC Group: five patients). Postoperative three‐dimensional (3D) double‐echo steady‐state (DESS) sequence with radial imaging at 3 Tesla were obtained and fluoroscopic image of the involved hip under distraction were used to observe the restoration of vacuum effect. Patient‐reported outcome scores (PROs) including the Harris Hip Score (HHS), Visual Analogue Score (VAS), Hip Outcome Score Activities of Daily Living Subscale (HOS‐ADL), Hip Outcome Score Sport‐Specific Subscale (HOS‐SSS) were collected and compared between two groups.

**Results:**

All patients were followed up for at least 6 months. The follow‐up time of RESEC group is longer than MICRO group (46.6 months *vs* 23.9 months, *P* < 0.05). The 3D DESS imaging demonstrated intermediate signal intensity at the relative area where the labrum resected followed by microfracture at the acetabular rim in MICRO group. Meanwhile, regrowth of labrum‐like tissue was not observed in MRI imaging of the RESEC group. Furthermore, vacuum effect was more apparent in MICRO group compared with RESEC group. All PROs in both groups showed a statistically significant improvement at follow‐up compared with preoperative levels. RESEC group: HHS (73.0 *vs* 93.8, *P* < 0.05); HOS‐ADL (51.5 *vs* 89.1, *P* < 0.05); HOS‐SSS (47.8 *vs* 88.3, *P* < 0.05); VAS (6.4 *vs* 2.0, *P* < 0.05). MICRO group: HHS (70.5 *vs* 91.5, *P* < 0.05); HOS‐ADL (52.4 *vs* 87.0, *P* < 0.05); HOS‐SSS (48.1 *vs* 86.5, *P* < 0.05); VAS (6.3 *vs* 1.6, *P* < 0.05). One patient of MICRO group had transient neurapraxias of the pudendal nerve that resolved completely by 3 months. There showed no statistically significant difference between groups regarding the preoperative and postoperative PROs.

**Conclusion:**

Compared to labral resection, combined arthroscopic labral resection and microfracture at the rim of acetabulum is able to fulfill the labral defect area with the potential to restore the seal effect of labrum as an effective and safe option for irreparable segmental labral tears.

## Graphical abstract

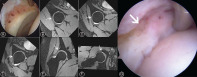

To our knowledge, no relative studies exist about utilizing microfracture as a therapy for irreparable labral tear. Through microfracture, outflowing undifferentiated stem cells is reported to have the capacity of recreating a fibrocartilaginous tissue histologically in the space among capsule, acetabulum and femur.

## Introduction

The labrum of hip is a triangular fibrocartilage attached to the acetabular rim almost circumferentially. The labrum's main function is to mainly create a vacuum with a negative pressure that increases the difficulty of dislocating the hip and retains the fluid within the central compartment to lubricate the joint and even the distribution of contact forces across the articular surface, preventing early arthritic wear^1^, ^2^. In addition, synovial fluid within the central compartment provides nutrition for the chondrocytes *via* diffusion preventing femoral and acetabular cartilage from degeneration. The labrum also deepens the acetabular articular surfaces, which is able to protect the femoral head from dislocating. A labral tear occurs most commonly under the circumstance of fermoroacetabular impingement, which is characteristic by deformity of acetabulum, femoral head or both^3^. A labral tear can cause anterior hip or groin pain with concomitant symptoms like clicking, popping and catching. If the torn labrum and the underlying etiology remains untreated, the hip joint may progress to osteoarthritis prematurely induced by the mechanisms of the absence of efficient synovial fluid friction and physiological stress environment of hip^2^, ^4^.

The acetabular labrum plays an important role in preventing a healthy hip from premature arthritis. The preservation of the hydraulic seal effect of labrum is closely associated with satisfactory clinical outcomes no matter what kind of surgical treatment is performed. Since the role of the labrum has been supported by evidence, resection of a serious torn labrum may progress to a chondral lesion and premature arthritis and disrupt the role of the labrum in proprioception associated with the mechanical pain. Labral repair is increasingly the favored option for many surgeons as the preservation of the labrum leads to a superior outcome compared with debridement of the labrum^5^, ^6^, ^7^. Although preferred by most surgeons, primary labral repair can be challenging. When the labrum is deemed irreparable due to a hypotrophy labrum or previous debrided labrum, despite the satisfied short‐term outcomes of labral resection in some research^8^, simple excision of a torn labrum accounts for an inferior outcome due to its loss of suction seal effect^6^, ^9^. Thus, an increasing number of surgeons intend to advocate labral reconstruction, which claims to have the ability of restoring labral seal effect^10^, ^11^, ^12^, ^13^. Nonetheless, enormous operating challenges and cost effectiveness aside, labral reconstruction requires accumulation of extensive surgical experience despite the superior postoperative outcome reported in various research^10^, ^14^, ^15^.

Despite the fact that postoperative outcome of labral reconstruction is reported to be good, the possibility of restoring the labral seal effect remains uncertain because of the conversion rate of THA after labral reconstruction. This may raise the controversy over whether labral reconstruction can restore the suction seal effect which is of great importance in preventing premature articular wear. Moreover, current follow‐ups of various research range from short to mid‐term. No long‐term outcome of clinical trials is reported to ensure the role of labral reconstruction. Thus, the current status of treatment of irreparable labral tear remains uncertain.

Under these circumstances, we wonder if there is another simple and effective treatment which is easy to operate. To our knowledge, no relative studies exist about utilizing microfracture as a therapy for irreparable labral tear. As the typical kind of marrow stimulation technique, microfracture is considered to be a first line of surgery for chondral defects because of its simplicity, cost‐effectiveness and low patient morbidity. Through penetrating the subchondral plate, the following marrow simulation brings the undifferentiated stem cells into the defect. Then a marrow clot forms at the defect area, which provides an environment for both pluripotential marrow cells and mesenchymal stem cells to differentiate into stable tissue within the base of the lesion. The regrowth tissue is able to replace the damaged cartilage and partially restore its function.

Therefore, through microfracture at the rim of acetabulum, outflowing undifferentiated stem cells is reported to have the capacity of recreating a fibrocartilaginous tissue histologically in the space among capsule, acetabulum and femur. Traditionally, the scar formed at the acetabular rim leading to severe postoperative adhesion is considered as the possible cause of recurrent hip pain. However, there exists no research to validate this standpoint. Thus, the functional outcome of labral resection alone was used as a comparison with that of the combined arthroscopic labral resection and microfracture at the rim of acetabulum. We hypothesize that fibrocartilage‐like tissue would grow at the labral defect, function as a gasket seal like labrum and cause no postoperative hip pain. In addition, the short‐term clinical outcome of combined arthroscopic labral resection and microfracture at the rim of acetabulum is expected to be superior to labral resection.

The purposes of this study were: (i) to assess the possibility of fibrocartilaginous repair in the labral defect area when performing microfracture at the acetabulum rim; (ii) to make sure the potential of regrowth tissue to restore hydraulic seal effect; and (iii) to find out whether the regrowth tissue is responsible for postoperative hip pain.

## Methods and Materials

This is a retrospective case–control study, which was approved by the Ethics Committee of First Affiliated Hospital of Army Medical University. Moreover, the informed consent was obtained from all included patients.

### Inclusion and Exclusion Criteria

Inclusion criteria: (i) patients presented with suspected anterior hip or groin pain with or without concomitant mechanical symptoms including clicking, popping and catching; (ii) the anterior hip or groin pain failed to relieve by conservative treatment for at least 3 month; (iii) magnetic resonance imaging findings of irreparable labral tear confirmed by intraoperative findings; (iv) anterior–posterior pelvis view radiographs showed no severe osteoarthritis (Tönnis grade 0, 1); and (v) patients underwent hip arthroscopy with a minimum of 0.5‐year of follow‐up.

Exclusion criteria: (i) patients with concomitant pathology like synovial chondromatosis, dysplasia hip, Perthes disease and acute trauma of hip; (ii) postoperative period less than 0.5 year; and (iii) patients undergoing microfracture of femoral head for severe chondral lesions. Patients who met one of the criteria mentioned above would be excluded.

Between 28 February 2014 and 26 February 2018, data on all patients undergoing primary hip arthroscopic surgery with the senior surgeon were retrospectively collected and reviewed. those patients included were diagnosed with fermoroacetabular impingement, among whom there were 17 cases of Pincer type, five cases of Cam type and three cases of mixed type. All hips that underwent arthroscopic labral resection with or without microfracture at the rim of acetabulum for 1 year were included in this study. Thirteen patients were excluded owing to patient refusal of participation and concomitant diseases like synovial chondromatosis or dysplasia hip. Patients were further assessed by radiographic tools preoperatively consisting of anterior–posterior pelvis, frog‐leg lateral view radiographs and magnetic resonance imaging. Irreparable labral tear was determined intraoperatively: severe intra substance damage, labral ossification, or segmental defects treated by either arthroscopic debridement alone or combined arthroscopic labral resection and microfracture at the rim of acetabulum. The total number of included hips were 30 from 25 patients (one side at a time for patients of bilateral labral tear at an interval of 0.5 year), who were divided into two groups: patients undergoing combined arthroscopic labral resection and microfracture at the rim of acetabulum (MICRO Group: 20 patients/23 hips) and labral resection alone (RESEC Group: five patients/seven hips).

### Operative Procedure

#### Anesthesia and Portal Placement

Patients were positioned supine on a traction table and placed under combined spinal and epidural anesthesia or general anesthesia. After assessment of hip joint, three portals were established: anterior, anterolateral and posterolateral portal. Anterior portal was placed at the intersection of a vertical line descending from the anterior‐superior iliac spine and a horizontal line from the tip of the greater trochanter. Anterolateral was placed 1 cm anterior and 1 cm superior to the tip of the greater trochanter. Posterolateral portal was placed 1 cm superior and 1 cm posterior to the tip of the greater trochanter.

#### Probing

After the portals were established, the labral tear was inspected and carefully probed. The labral tear extent was described using a clock‐face pattern, with 3 o'clock always indicating anterior.

#### Management of Irreparable Labral Tears

**Fig. 1:**
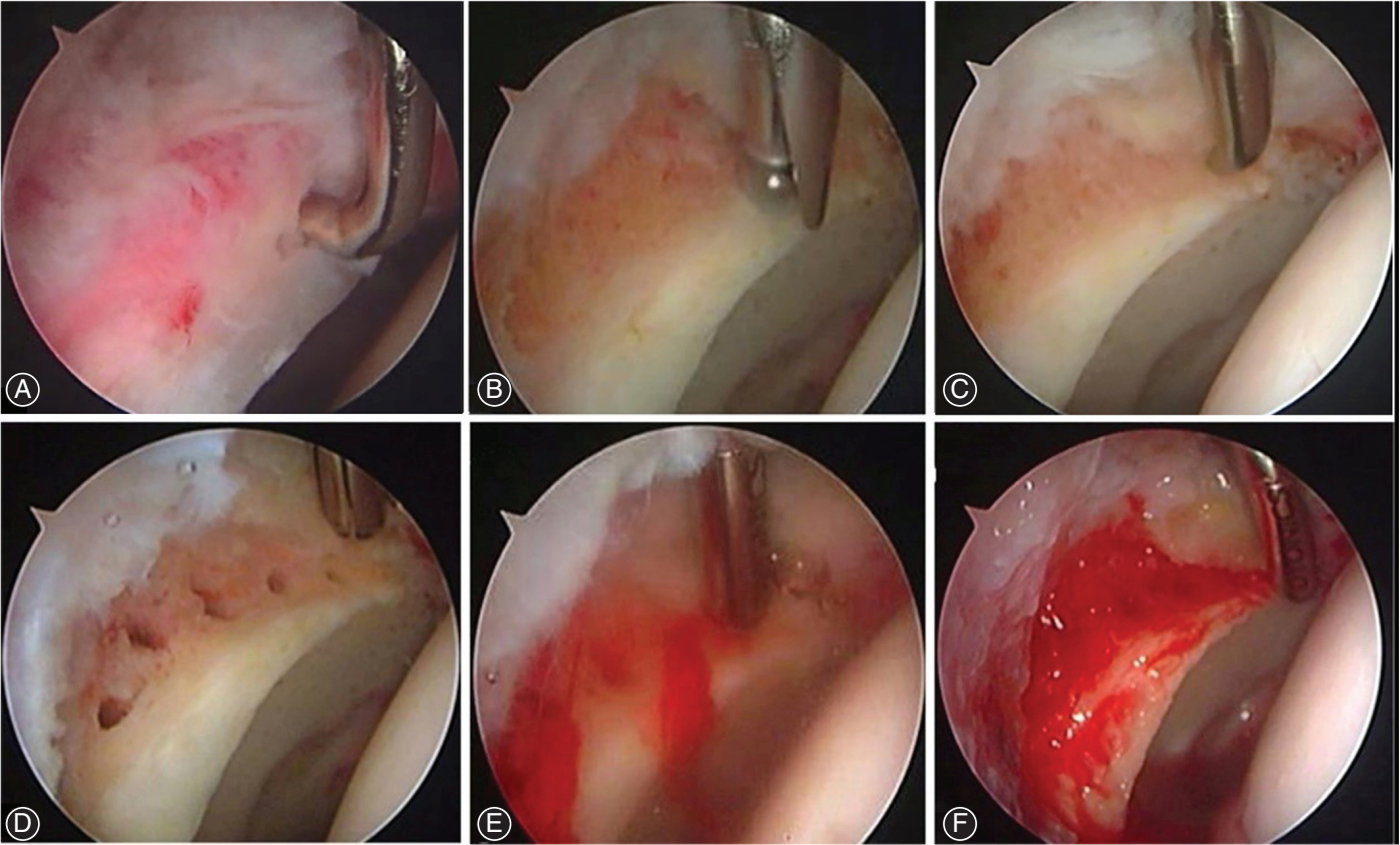
Procedures of combined arthroscopic treatment of labral resection and microfracture at the rim of acetabulum: (A) Labral debridement. (B) Acetabuloplasty with a burr. (C) Microfracture using an awl. (D) Complement of microfracture. (E) Marrow bleeding. (F) Blood clot forming in the labral defect.

**Fig. 2:**
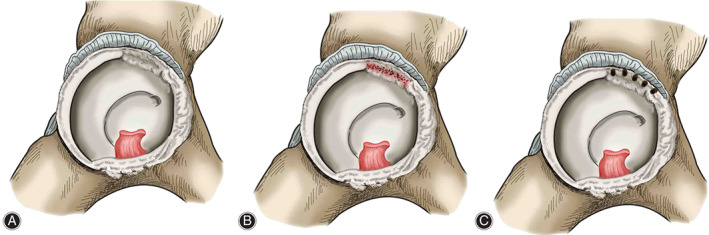
Schematic diagram of critical surgical procedures: (A) Labral resection. (B) Dotted bleeding after acetabuloplasty with a burr. (C) Multiple small holes (2 mm in diameter) spaced 3–4 mm apart is made as microfracture with an awl.

If the injured labrum was deemed irreparable, then it was completely debrided followed by the procedure that the acetabular rim was freshened for a bleeding bed, and/or a correction of Pincer deformity was performed if existed. When performing microfracture at the rim of acetabulum, a surgical awl (Smith & Nephew, Andover, MA, USA) is used to make multiple small holes (2 mm in diameter) spaced 3–4 mm apart representing a linear shape in the exposed bone of the labral defect. Finally, sufficient marrow bleeding flowing from the small hole is observed after turning off arthroscopic pump (Smith & Nephew, Andover, MA, USA) (Figs 1, 2).

#### Management of Concomitant Pathology

Iliopsoas releasing was performed if there existed painful internal snapping hip syndrome preoperatively (two cases). Concomitant pathology like severe acetabular chondral lesions were addressed with microfracture, and ligamentum teres tears were treated with debridement (one case). Bony prominence of the femoral neck (cam deformity) was resected using a bur (Smith & Nephew, Andover, MA, USA), and any loose bodies observed were removed. The capsule was not closed after interportal capsulotomy was performed.

#### Postoperative Management

Postoperatively, all patients were instructed to toe‐touching weight bearing for 2 weeks and then transited from partial to full weight bearing for the following 4 weeks. In MICRO group, all patients used a hip brace to maintain slight flexion and external rotation to 0° of the involved hips for 2 weeks with limited motion of hip flexion within 60° in the next 4 weeks. In addition, physical therapy was performed on postoperative day one mainly focusing on straight leg raise training, which is supposed to last 6 weeks.

### Radiographic Evaluation

**Fig. 3:**
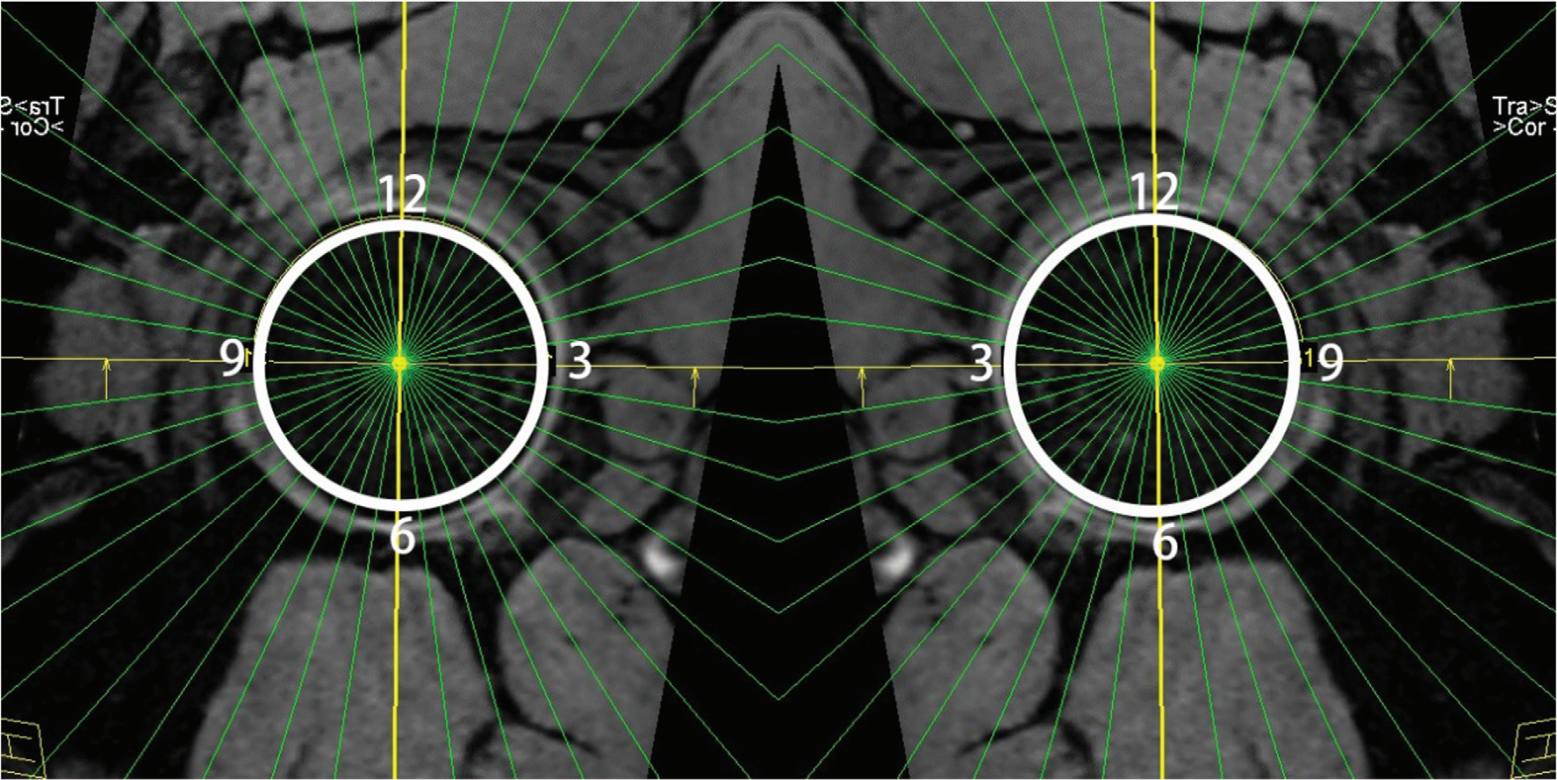
The radial imaging improves the visualization of labrum and provides information on the location of labrum (i.e. 3 o'clock to12 o'clock referring to anterior to superior).

All patients were evaluated with three‐dimensional (3D) double‐echo steady‐state (DESS) reformatted magnetic resonance images (MRI) (Magneton Spectra, Siemens, Berlin, Germany). Radial reformatted images of axial 3D DESS sequence was reconstructed at 6° slice intervals through the center of the acetabulum perpendicular to the plane across the entire acetabular rim. A clock‐face scheme from 3 o'clock to 9 o'clock was implemented to describe the location of labrum (Fig. 3). Among those images, the labral defect area was located according to the performed arthroscopy video and the signal intensity at the acetabular rim was recorded.

### Evaluation of Functional Outcomes

At latest follow‐up, all patients were assessed using Harris Hip Score (HHS), Visual Analogue Score (VAS), Hip Outcome Score Activities of Daily Living Subscale (HOS‐ADL), Hip Outcome Score Sport‐Specific Subscale (HOS‐SSS) to determine hip function outcome. Moreover, fluoroscopic image of the involved hip under distraction was used to evaluate the vacuum effect.

#### Harris Hip Score (HHS)

The HHS was used to evaluate postoperative recovery of hip function in an adult population. The HHS score system mainly includes four aspects as pain, function, absence of deformity, and range of motion. The score standard had a maximum of 100 points (best possible outcome). A total score <70 is considered a poor score, 70–80 fair, 80–90 is good and 90–100 excellent.

#### Visual Analog Scale (VAS)

The VAS is a well described pain scale whereby patients report their pain from 0 to 10. A score of 0 would indicate no pain and 10 would indicate the worst pain. The VAS allows patients a simple method of relaying their experience regarding how painful their condition is. Patients are asked to rate their pain on a 0–10 scale. The clinical significance of this is that it allows patients to compare their own pain based on their perception of their own previous pain. It also objectifies the pain level to allow for quantitative analysis. Patients are generally more functional with less pain.

#### Hip Outcome Score Activities of Daily Living Subscale (HOS‐ADL) and Sport‐Specific Subscale (HOS‐SSS)

The Hip Outcome Score (HOS) was specifically developed for younger more active patients between the ages of 13 and 66 years. The HOS is a patient‐administered tool that was designed to assess self‐reported functional status; therefore, symptoms were not considered part of the functional assessment. The HOS includes two subscales: activities of daily living (ADL) and sports‐specific (SSS). The scoring of the HOS is somewhat complicated since each subscale is scored separately as a percentage score. The ADL subscale has 19 items but only 17 are scored. The items pertaining to sitting and putting on socks and shoes are not included. The sports subscale has nine items. Each item on both scales is scored from 4 to 0, with 4 indicating “no difficulty” and 0 indicating “unable to do.” There is a “not applicable” option as well. The percentage score is calculated by comparing the item total score divided by the highest potential score multiplied by 100. By design the HOS does not represent a true patient‐derived outcome since it does not include items of specific concern to patients such as symptoms and work‐related, social, or emotional issues. It should be considered a well‐designed and evaluated functional outcome measure.

### Statistical Analysis

All statistical analyses were performed with SPSS software (version 21, SPSS Inc., Chicago, IL, USA), and a *P* value <0.05 was considered statistically significant. Comparisons of postoperative patient‐reported outcome scores (PROs) and demographic statistics between the labral treatment groups were performed with 2‐tailed Student *t* tests.

## Results

### General Results

There were no significant differences in comparison of age (42.7 *vs* 44.6 years), BMI (Body Mass Index) (22.5 *vs* 22.8) between MICRO and RESEC group (Table 1). The follow‐up time is longer in RESEC group (46.6 months *vs* 23.9 months, *P* < 0.05).

**TABLE 1:** Demographics for microfracture and resection group

Demographics	Microfracture group	Resection group	Statistic value	*P* value
Age (years)	42.7 ± 12.1	44.6 ± 9.4	*t* = 0.325	0.748
Sex (male/female)	8/12	4/1		0.160
BMI (kg/m^2^)	22.5 ± 2.7	22.8 ± 2.5	*t* = 0.202	0.842
Follow‐up (months)	23.9 ± 7.5	46.6 ± 5.4	*t* = 6.369	<0.001
Involved hips	23	7		
Location of labral defect				<0.05
Superior	3	0		
Anterior	1	0		
Anterosuperior	19	7		
Preoperative outcome score				
HHS	70.5 ± 5.5	73.0 ± 2.1	*t* = 1.601	0.127
HOS‐ADL	52.4 ± 5.4	51.5 ± 2.3	*t* = 0.385	0.704
HOS‐SSS	48.1 ± 7.7	47.8 ± 5.4	*t* = 0.077	0.940
VAS	6.3 ± 1.0	6.4 ± 1.1	*t* = 0.190	0.851
Postoperative outcome score				
HHS	91.5 ± 6.5	93.8 ± 2.5	*t* = 0.770	0.449
HOS‐ADL	87.0 ± 2.3	89.1 ± 2.6	*t* = 1.839	0.079
HOS‐SSS	86.5 ± 6.3	88.3 ± 2.2	*t* = 0.589	0.562
VAS	1.6 ± 0.9	2.0 ± 1.0	*t* = 0.885	0.385

### MRI Outcomes

**Fig. 4:**
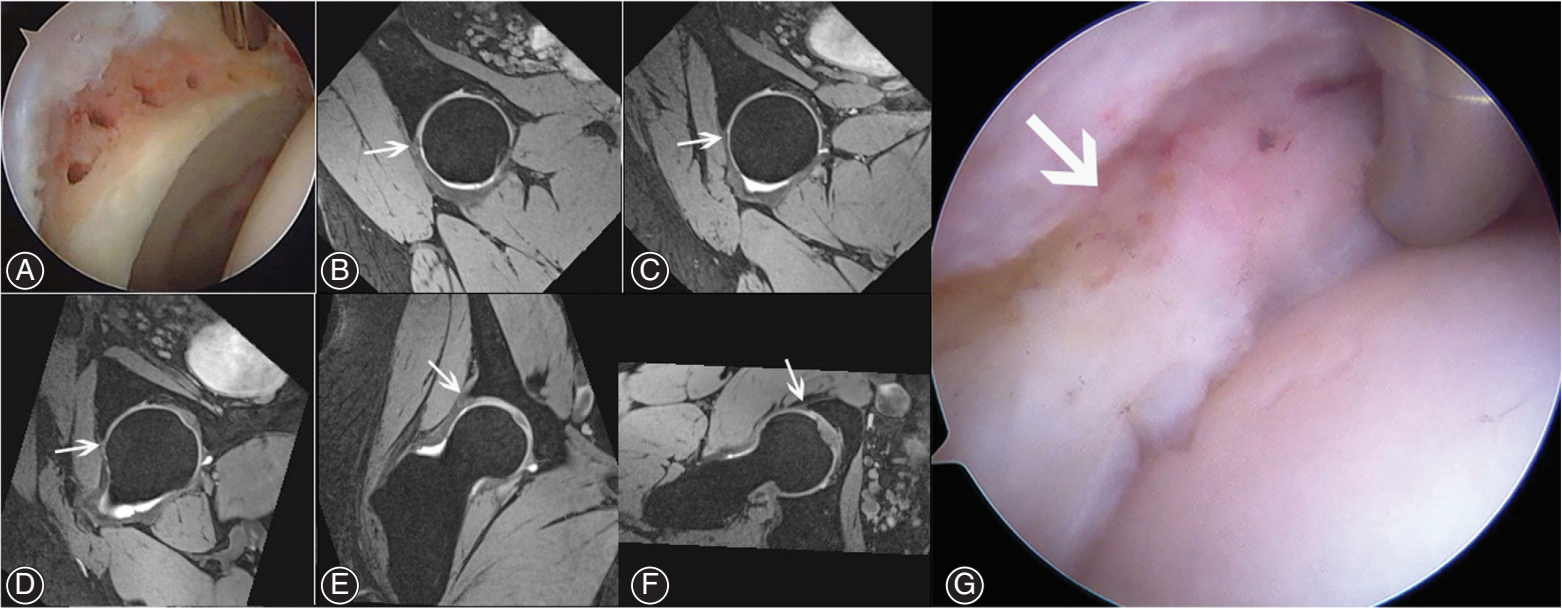
(A) Microfracture at the anterolateral acetabular rim after labral debridement. (B, C, D) Homogenous low signal (arrow) at the labral defect area in 1 o'clock, 12 o'clock and 11 o'clock of radial imaging. (E, F) Coronal and cross section showed the same homogenous low signal (arrow). (G) Regrowth tissue (arrow) was shown in second‐look arthroscopy.

**Fig. 5:**
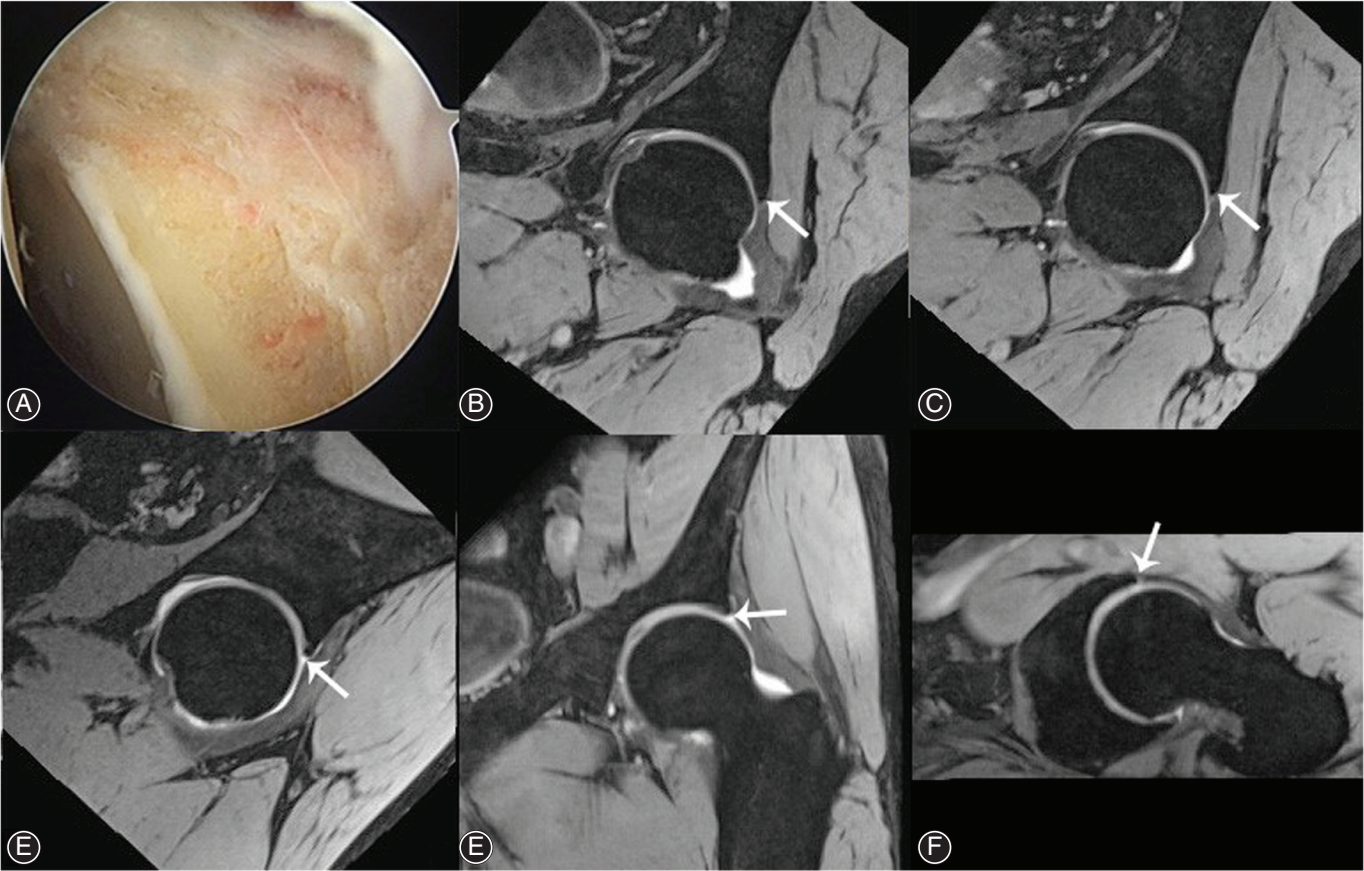
(A) Labral debridement without microfracture. (B, C, D) No obvious homogenous low signal was observed (arrow) at the labral defect area in 1 o'clock, 12 o'clock and 11 o'clock of radial imaging. (E, F) Coronal and cross section also showed no homogenous low signal (arrow).

At last follow‐up, 3D DESS MRI of all 23 hips showed recreation of fibrocartilage tissue along the acetabular rim where microfracture was performed while labral regrowth was not observed in those from the resection group (Figs 4 and 5). The location of labral defect area was summarized in Table 1. The integrality of subchondral bone plate was restored and bone tissue overgrowth into labral defect area was not observed in patients of both groups. There was also no evidence supporting the formation of subchondral cyst or bone marrow edema in any patients within the follow‐up.

### HHS, HOS and VAS Score

There was no statistically significant difference between groups in regard to the preoperative and postoperative PROs, or any of the demographic data (Table 1).

#### HOS

There was significant improvement between preoperative and postoperative HOS‐ADL and HOS‐SSS in both groups (Table 2). The mean change in the HOS‐ADL was 34.6 ± 5.6 in the MICRO group and 37.6 ± 4.8 in the RESEC group.

**TABLE 2:** Functional outcomes comparing of MICRO and RESEC group (mean ± SD)

PROs	Microfracture group				Resection group			
	Preoperation	Final follow‐up (8–39 months)	*t* value	*P* value	Preoperation	Final follow‐up (43–56 months)	*t* value	*P* value
HHS	70.5 ± 5.5	91.5 ± 6.5	18.199	<0.001	73.0 ± 2.1	93.8 ± 2.5	12.343	<0.001
HOS‐ADL	52.4 ± 5.4	87.0 ± 2.3	27.702	<0.001	51.5 ± 2.3	89.1 ± 2.6	17.509	<0.001
HOS‐SSS	48.1 ± 7.7	86.5 ± 6.3	24.361	<0.001	47.8 ± 5.4	88.3 ± 2.2	58.723	<0.001
VAS	6.3 ± 1.0	1.6 ± 0.9	20.385	<0.001	6.4 ± 1.1	2.0 ± 1.0	5.047	0.007

HHS, Harris Hip Score; HOS‐ADL, Hip Outcome Score Activities of Daily Living Subscale; HOS‐SSS, Hip Outcome Score Sport‐Specific Subscale; PROs, Patient‐reported outcome scores; VAS, Visual Analog Scale.

The mean change in the HOS‐SSS was 38.5 ± 7.1 in the MICRO group and 40.6 ± 1.5 in the RESEC group. There was no statistically significant difference between the mean changes in all PROs, despite having more improvement in scores in the RESEC group.

#### HHS and VAS

There was significant improvement between preoperative and postoperative HHS and VAS in both groups (Table 2).The mean change in the HHS was 21.0 ± 5.2 in the MICRO group and 20.8 ± 3.8 in the RESEC group.

The mean change in the VAS was 4.7 ± 1.0 in the MICRO group and 4.4 ± 1.9 in the RESEC group. There was no statistically significant difference between the mean changes in all PROs, despite having more improvement in scores in the MICRO group.

#### Vacuum Effect

**Fig. 6:**
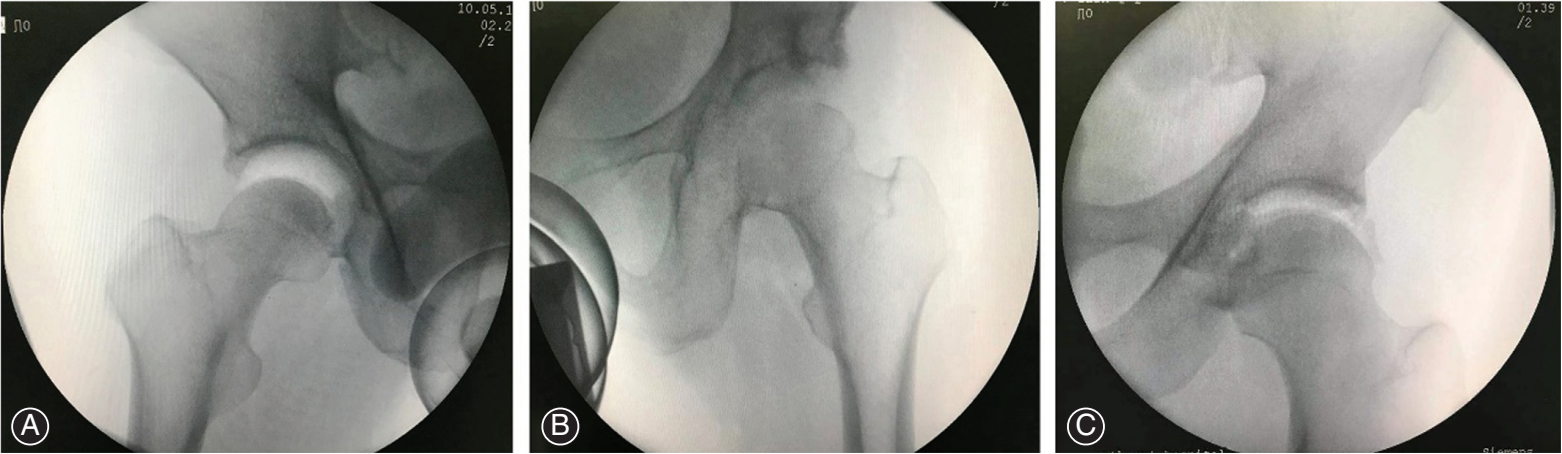
All patients underwent fluoroscopic image of the involved hip under distraction without anesthesia to evaluate the vacuum effect at the last follow‐up (an interval more than 0.5 year). (A) Apparent vacuum effect in Microfracture group. (B) Total loss of vacuum effect in Resection group. (C) Partial loss of vacuum effect in Resection group.

At last follow‐up, vacuum effect was found in every patient of MICRO group when performed the anterior–posterior view of the involved hip under distraction without anesthesia (Fig. 6). For RESEC group, partial or even total loss of vacuum effect (three hips) was observed in patients treated by labral resection.

### Complications

There were no major postoperative complications (e.g., femoral neck fractures, osteonecrosis, deep infections, pulmonary embolism, abdominal compartment syndrome). One patient in the MICRO group had transient neurapraxias of the pudendal nerve that resolved completely by 3 months. There were no sciatic, femoral, or lateral femoral cutaneous neurapraxias. There were no revision surgeries or conversions or scheduled conversions to resurfacing hip arthroplasty or total hip arthroplasty (THA) in both groups.

## Discussion

The DESS MRI provide the compelling evidence that the acetabular rim was covered with regrown tissue in every patient of microfracture group. Comparing with RESEC group, suction seal effect was restored proven by the apparent vacuum effect in all fluoroscopic view of patients from MICRO group. The short‐term clinical outcome of combined arthroscopic labral resection and microfracture at the rim of acetabulum is similar to labral resection.

### Current Status of Treatment of Irreparable Acetabular Labral Tear

In the case where the labrum is deemed irreparable because of insufficient mass, reconstruction either in open surgery or under arthroscopy is indicated with an autograft or allograft being incised to replace the torn labrum. Despite the fact that postoperative outcome of labral reconstruction is reported to be good, the possibility of restoring the labral seal effect remains uncertain. As far as we are concerned, the conversion rate of THA after labral reconstruction ranges from 0% to 25% reported in various research^5^, ^15^, ^16^, ^17^, ^18^, ^19^, ^20^, ^21^, ^22^, ^23^. With a minimum 1‐year follow‐up, Philippon *et al*. reported four of 47 patients had been converted to THA, three of those had a joint space of <2 mm thus indicated that joint space of <2 mm may be a risk factor for an inferior outcome for labral reconstruction^5^. Similar studies are published to support this statement^18^, ^20^. However, recent research by White *et al*. showed that 13 of 131 hips undergoing labral reconstruction progressed to arthritis requiring THA without preoperative joint space of <2 mm and Tönnis Grade ≥2^15^. Other studies have also indicated the similar outcome in patients showing Tönnis Grade 0 or 1 in preoperative radiograph^19^, ^21^. This may raise controversy over whether labral reconstruction can restore the suction seal effect which is of great importance to prevent a premature articular wear. In addition, long‐term study is needed to ensure the role of labral reconstruction.

### Tissue Regrowth of Microfracture at the Acetabular Rim

As the typical kind of marrow stimulation technique, microfracture is considered to be a first line of surgery for chondral defect because of its simplicity, cost‐effectiveness and low patient morbidity. Through penetrating the subchondral plate, the following marrow simulation brings the undifferentiated stem cells into the defect from the marrow forming a marrow clot, which provides an environment for both pluripotential marrow cells and mesenchymal stem cells to differentiation into stable tissue within the base of the lesion^24^. It is reported to have the capacity of recreating a fibrocartilaginous tissue histologically covering the previous lesion^25^. Although there does exist studies reporting poor repair volume on MRI and exposed bone on second‐look arthroscopy^26^, ^27^, a possible explanation for lack of filling is that the initially formed blood clot is displaced from the defect^28^. Furthermore, labral lesion frequently lies in the anterosuperior area where the labrum is close to the anterior capsule thus the narrow space between the exposed acetabular rim and capsule makes the clot probably unable to displace. On the other hand, the quality and longevity of the repair tissue has been doubted^29^, ^30^, which is quite reasonable as the fibrocartilage tissue contains more type I collagen that is vulnerable to stress. However, as acetabular labrum has little weight‐bearing function and mainly consists of type Icollagen^31^, the repair tissue at the acetabular rim seems to be able to survive the low‐stress condition. Compared with microfracture, the exposed bony rim of the acetabulum shown in DESS MRI of the resection group suggests that the labrum was unable to regrowth spontaneously. Interestingly, Abrams *et al*.^32^ stated that the labrum had the potential of regeneration after debridement supported by the second‐look arthroscopy in 21 hips. Nonetheless, the finding was questioned by Domb for the reason that the definition of boundary between the labrum and scar tissue was controversy such that the existence of regeneration remained uncertain^16^.

MRI is used for an evaluation for chondrolabral injuries but standard MRI produces both false‐positive results and an underestimation of labral pathology and has only 30% sensitivity and 36% accuracy^33^. Meanwhile, the technique of Magnetic Resonance Angiography is invasive and patients are probably under the risk of gadolinium contrast agent poisoning. Fitting all these considerations together, we have determined to use 3D DESS sequence with radial imaging at 3.0 Tesla to evaluate the labrum, the validity of which has been testified^34^. The visualization of the labrum and the cartilage is further improved due to the radial imaging approach^35^, ^36^. Based on the previous studies supporting the possibility of fibrocartilage repair and the high accuracy of DESS technique for detecting labral lesion, we believe that the intermedia signal intensity demonstrated by 3D DESS MRI is convincingly the results of fibrocartilage‐like tissue through marrow stimulation at the labral defect area.

### Potential of Labral Functional Restoration

It has been pointed out that the association exists between labral lesions and the onset of osteoarthrosis due to the alterations of hip joint biomechanics^2^, ^4^. An intact labrum is able to seal a pressurized layer of synovial fluid within the joint for joint lubrication and load support^2^, ^37^. A human cadaveric research by Ferguson et al. demonstrated a 21% increase in steady state cartilage consolidation after labral resection due to the lack of pressurized fluid, leading to a direct solid‐on‐solid contact^2^. Therefore, the sealing function of the labrum is of great importance to hip stability as it provides a low friction articulation and even load distribution during joint motion. However, the intra‐articular fluid pressure produced by labrum sealing was measured with transducers inserted into the central compartment of cadaveric hip in previous studies, which is difficult to implement in our follow‐up study. To our knowledge, no center has focused their follow‐up on the restoration of labrum function other than functional scoring. Under this circumstance, we consider vacuum phenomenon as an indirect method to observe the restoration of labral seal effect. The vacuum phenomenon is caused by the negative intracapsular pressure during distraction^38^. Interestingly, the capsules in all hips undergoing interportal capsulotomy were not closed and the fluoroscopic outcomes of MICRO group still demonstrated apparent vacuum effect. Although the capsule could be repaired by scar tissue, it may imply that the restoration of labral seal is implicated in the existence of the vacuum phenomenon. With the goal of detecting the effect of labral tear on the vacuum effect, we examined a patient who had undergone THA because of severe osteoarthritis with fluoroscopic image of the involved hip under distraction preoperatively. It turned out that the vacuum effect was absent while the anterosuperior labrum was found to be torn intraoperatively. For all we know, no research has been reported about the factors that influence the production of vacuum effect, which needs to be explored in the future. At least our fluoroscopic results might hint that the regrown tissue has the chance in helping restore the hydraulic seal effect.

### Limitations

Limitations of our study include lack of pathology results in second‐look hip arthroscopy and small sample size of the resection group. As the procedure of microfracture to treat irreparable torn labrum shortly came up, after which labral excision was no longer an option for irreparable labral lesions, the number of patients undergoing labral resection before that is relatively small. The observation of the vacuum effect is a qualitative approach which is not able to define the difference of the restoration of negative pressure between the two groups quantitatively. Whether the unfixed capsule of our patients will have an impact on the restoration of the vacuum effect remains uncertain. Long‐term follow‐up is expected to further explore the clinical outcomes.

To sum up, comparing with labral reconstruction, combined arthroscopic treatment of labral resection and microfracture at the rim of acetabulum is an easy, safe and effective procedure which is suitable for segmental irreparable labral tear as partial tissue regrowth could be achieved after marrow stimulation. When faced with the dilemma that labral reconstruction could not be performed, microfracture might be a cost‐effective and efficient option.

### Conclusions

Through effective marrow stimulation at the acetabular rim, the labral defect is repaired by the regrown tissue. Most importantly, the repaired tissue is able to restore the hydraulic seal effect of the labrum. Further study is needed to determine the long‐term clinical outcomes.
